# Effects of infrared laser moxibustion on cancer-related fatigue in breast cancer survivors: Study protocol for a randomized controlled trial

**DOI:** 10.1097/MD.0000000000016882

**Published:** 2019-08-23

**Authors:** Huijuan Mao, Jun J. Mao, Junchao Chen, Qing Li, Xuefen Chen, Xubo Shen, Ling Zhao, Jianzi Wei, Xueyong Shen

**Affiliations:** a Shanghai University of Traditional Chinese Medicine, School of Acupuncture-Moxibustion and Tuina, Shanghai, China; b Memorial Sloan Kettering Cancer Center, Bendheim Integrative Medicine Center, New York, NY; c Shanghai University of Traditional Chinese Medicine, Institute of Disciplinary Science; d Shanghai University of Traditional Chinese Medicine, School of Public Health; e Shanghai University of Traditional Chinese Medicine, Yueyang Hospital of Integrated Traditional Chinese and Western Medicine; f Shanghai Research Center of Acupuncture and Meridian, Shanghai, China.

**Keywords:** acupuncture, breast cancer, cancer survivor, cancer-related fatigue, infrared laser, moxibustion

## Abstract

**Background:**

Cancer-related fatigue (CRF) is the most common and distressing symptom associated with cancer treatment that breast cancer survivors (BCS) experience. We previously found that laser moxibustion may be efficacious for CRF. The primary aim of this study is to determine the specific efficacy of 10.6 μm infrared laser moxibustion on CRF. The secondary aim is to evaluate the effect of infrared laser moxibustion on co-existing symptoms that BCS experience.

**Methods:**

We will conduct a randomized, sham-controlled, three-arm trial of infrared laser moxibustion (ILM) against sham ILM (SILM) and waitlist control (WLC) among BCS with moderate to severe fatigue. The two intervention groups will receive either real or sham infrared laser moxibustion on four acupoints (i.e., ST36 [bilateral], CV4, and CV6) for 20 minutes each session for 6 weeks (twice per week). The primary endpoint is the change in fatigue score from Baseline to Week 6 as measured by the Chinese version of the Brief Fatigue Inventory (BFI-C). Our secondary aim is to compare the severity of co-morbidities (e.g., depression, insomnia, and pain) among the 3 groups.

**Discussion:**

The results of our trial will establish evidence for the efficacy of infrared laser moxibustion for CRF, a very common and challenging symptom.

**Trial registration number:**

NCT03553355.

## 1 Introduction

Cancer-related fatigue (CRF) is the most common and distressing symptom associated with cancer treatment. The prevalence of CRF ranges from 25% to 99% during active cancer treatment^[1,2]^ and it can persist for five to ten years after completion of treatment in approximately 30% of cancer survivors.^[3]^ Breast cancer is the most common cancer in women worldwide. Because of early detection and effective treatment, breast cancer survivors (BCS) constitute the largest population of cancer survivors.^[4,5]^ CRF contributes to impaired functioning and a decline in overall quality of life,^[6,7]^ and is a significant predictor of recurrence-free survival in BCS.^[8]^ Hence, the management of CRF to improve the quality of life in BCS is critically important.

Despite high levels of clinical significance, managing CRF is challenging.^[9]^ The effectiveness of pharmacologic treatments for CRF, including erythropoietin (e.g., epoetin, darbepoetin), psychostimulants (e.g., methylphenidate, modafinil), and anti-inflammatories (e.g., corticosteroids) is tenuous, and negative side effects are common and can be debilitating.^[10,11]^ Recently, integrative medicine therapies have emerged as promising non-pharmacological treatment options many cancer survivors use to reduce CRF.^[12,13]^ Further, a current systematic review suggests exercise or psychological interventions as first-line treatments for CRF.^[14]^ However, fatigue is a major barrier to starting or maintaining physical activity among cancer patients.^[15,16]^

Growing evidence suggests that acupuncture, a common integrative medicine treatment, may be safe and effective for managing CRF^[17–19]^ When it is delivered by adequately trained practitioners, acupuncture is generally considered to be a safe form of treatment.^[20,21]^ Moxibustion, a modality of acupuncture, is a noninvasive procedure that involves burning moxa, the herb *Artemisia vulgaris*, on or above the skin at acupoints. Instead of using the needle stimuli of acupuncture, moxibustion provides heat stimuli to alleviate symptoms; this method may be particularly appealing to patients who do not like the needle penetration associated with acupuncture.^[22]^ To our knowledge, research concerning the effects of moxibustion on CRF has only been reported in a few Chinese language articles and most of the studies had a high risk of bias, such as small sample size or no sham control.^[23]^ Furthermore, traditional moxibustion has several shortcomings, including air pollution and potential burning of the skin.

Infrared laser moxibustion, a novel noninvasive and painless therapy, mimics the effect of traditional moxibustion to irradiate on acupoints with a 10.6 μm infrared laser. It avoids the shortcomings of traditional moxibustion, such as smoke, unpleasant smell, and difficulty in controlling the dosage. Our previous study demonstrated that the specific wavelengths of infrared radiation produced by moxibustion were as potent as those generated by thermal radiation and that the peak wavelength of infrared radiation from conventional partitioned moxibustion was approximately 10 μm.^[24]^ In our preliminary study (N = 61), infrared laser moxibustion appeared to be safe and efficacious for improving CRF in a Chinese patient population.^[25]^ Building on this promising pilot data, we designed the current trial to evaluate the specific efficacy of infrared laser moxibustion on CRF in BCS.

## 2 Specific aims and hypotheses

Our primary specific aim is to evaluate the efficacy of infrared laser moxibustion on CRF in BCS. We hypothesize that the Infrared Laser Moxibustion (ILM) group will have significantly greater improvement in the fatigue score from Baseline to Week 6 (primary end point) to Week 18 (follow up) compared to sham ILM (SILM) and waitlist control (WLC) groups. The primary outcome measure is the change over time in the mean score of the Chinese version of the Brief Fatigue Inventory (BFI-C).

Our secondary aim is to evaluate the efficacy of infrared laser moxibustion in insomnia, anxiety, depression, and pain among BCS experiencing CRF. We hypothesize that BCS with CRF randomized to ILM will have significantly greater improvement in insomnia, anxiety, depression, pain, and quality of life from Baseline to Week 6 and from Baseline to Week 18 compared to those randomized to SILM and WLC. Secondary outcome measures include the change over time in the mean score of the Chinese versions of the Pittsburgh Sleep Quality Index (PSQI), Hospital Anxiety and Depression Scale (HADS), Brief Pain Inventory (BPI), and Functional Assessment of Cancer Therapy-Breast (FACT-B).

Our exploratory mechanistic aim is to explore the effects of infrared laser moxibustion on telomere length among BCS with CRF. We hypothesize that BCS with CRF who are randomized to ILM will have a significantly greater increase in telomere length over time from Baseline compared to SILM and WLC.

## 3 Methods

### 3.1 Study design

**Figure 1:**
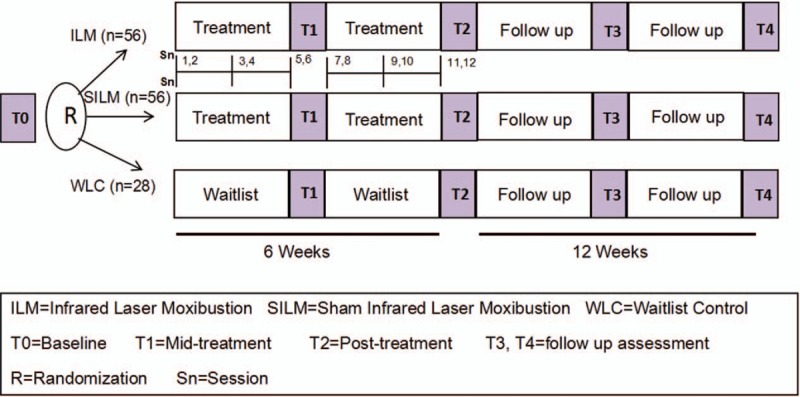
Study timeline.

We will conduct a randomized controlled, 3-arm trial of ILM compared to SILM and WLC for moderate (score of 4–6 on BFI-C) to severe (score of 7–10) fatigue in BCS. After random assignment to 3 groups, patients in the ILM and SILM groups will receive 20-minute treatments of real/sham infrared laser moxibustion twice per week for six weeks. We will have a 12-week follow-up period to evaluate the durability of the treatment effects. We will assess patient-reported fatigue and other comorbid symptoms at Baseline, Weeks, 6, 12, and 18, respectively. We have included details of the study timeline in Figure 1. This study was approved by the Institutional Review Board of Yueyang Hospital and registered with the identifier NCT03553355 (12 June 2018) at ClinicalTrials.gov. All participants will provide written informed consent.

Recruitment started in June 2018 and will continue until December 2019. We will follow the Consolidated Standards of Reporting Trials (CONSORT) guidelines for reporting on nonpharmacological treatment interventions^[26]^ and the Standards for Reporting Interventions in Clinical Trials of Acupuncture (STRICTA).^[27]^

### 3.2 Participants

Eligible study participants will be female breast cancer survivors (stage I–III), aged 18 years and older, who are at least 12 weeks post primary treatment (e.g., surgery, chemotherapy, and radiotherapy) and have complained of persistent, moderate to severe fatigue despite having the ability to rest. We will interview participants to ensure that they meet the International Classification of Diseases-10 diagnostic criteria for CRF.^[28]^ We will screen participants using the National Comprehensive Cancer Network (NCCN) clinical guidelines for CRF^[29]^ and an item asking respondents to rate their level of fatigue over the past seven days on a scale of 0 (“no fatigue”) to 10 (“worst fatigue you can imagine”) with a score of 4 as the cutoff point. We will exclude treatable etiologies (e.g., anemia and hypothyroidism). We have detailed our eligibility criteria along with the source of that material and the study personnel responsible for determining that criteria in Table 1

**Table 1:**
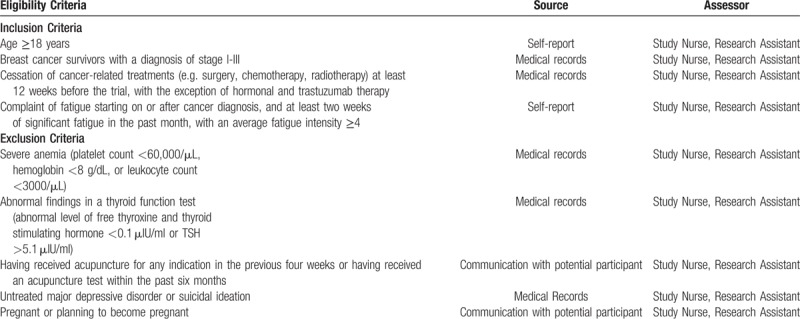
Eligibility criteria and source document.

### 3.3 Recruitment and enrollment

We will recruit patients from oncology clinics at Yueyang Hospital affiliated with the Shanghai University of Traditional Chinese Medicine in Shanghai, China. We will post clinical trial information on notice boards in this hospital and release recruitment notices on flyers and social networks. We will also contact the oncology physicians for referral of their potentially eligible patients. Then we will contact the potential participants via telephone triage and interview them to determine final and overall eligibility. We will conduct and obtain informed consent at the first interview. Following consent, we will collect baseline patient-reported outcomes and clinical data.

### 3.4 Randomization and allocation concealment

We will randomize participants to ILM, SILM, or WLC groups with an allocation ratio of 2:2:1. The biostatistician will conduct allocation using permuted block randomization with varying block sizes, stratified by age (<50/≥50 years of age) and baseline fatigue level (<7/≥7). The randomization sequence generation will be completed prior to the recruitment of participants and the investigators will remain blinded to the treatment condition. Randomization information will be sealed in opaque envelopes that we will open after participants complete their baseline assessment.

### 3.5 Interventions

#### 3.5.1 Infrared laser moxibustion therapy

We will use SX10-C1 laser moxibustion devices (Shanghai Wonderful Opto-Electrics Tech Co. Ltd., Shanghai, China) for the ILM and SILM groups. Four laser probes will be simultaneously aligned with four points (ST36 [bilateral], CV4, and CV6 acupoints) and we will irradiate each acupoint 2 cm away from the skin surface for a total of 20 minutes. Each patient will receive this treatment twice per week for six weeks (12 session total).

#### 3.5.2 Sham infrared laser moxibustion therapy

Patients in the SILM group will adhere to the same treatment protocol as those in the ILM group. The sham laser moxibustion instrument appears to be identical to the real one. However, in the sham group, no laser will be released when the instrument is turned on. Because the infrared laser is colorless, neither the operator nor the patients can see it, so the procedure will be double-blinded.

#### 3.5.3 Waitlist control

The participants in the WLC group will not receive real or sham laser moxibustion treatment. They will maintain their usual treatment and self-care, but will not begin any additional treatment to improve their cancer-related fatigue during the study. In addition, we will inform them that they will receive ten real laser moxibustion treatments after completing the study follow-up period.

We will not restrict medications study participants may use. We will enter patients’ previous medications in the database. In the event that during the study a patient starts a new regimen that might affect their fatigue severity, we will do sensitivity analyses to exclude this participant.

### 3.6 Masking

The principal investigator, study investigators, laser instrument operator, participants, outcome assessors, and biostatistician will all be blinded to the treatment assignments between real and sham laser moxibustion. Only outcome assessors and the statistician will be blinded as to whether the patient receives real/sham laser moxibustion or waitlist control.

The laser instrument for the sham group is designed so that the participants will not receive complete warmth at the site of application. While some participants in the ILM group will feel warm, these 2 groups will be treated separately so that they will not have a chance to communicate. At the end of the intervention period, participants and operators in ILM or SILM will assess the masking. They will be asked to guess their group assignment.

### 3.7 Outcome measures

Outcome measures and data collection time points are listed in Table 2. The primary endpoint of this study is the post-intervention assessment (T2, after 6 weeks of treatment sessions). We will measure primary and secondary outcomes at baseline (T0), mid-intervention (T1, after 3 weeks of treatment sessions), post-intervention (T2) and at follow-up (T3 and T4, 6, and 12 weeks after 6 weeks of treatment sessions). The collected clinical data will be collected with REDCap.

**Table 2:**
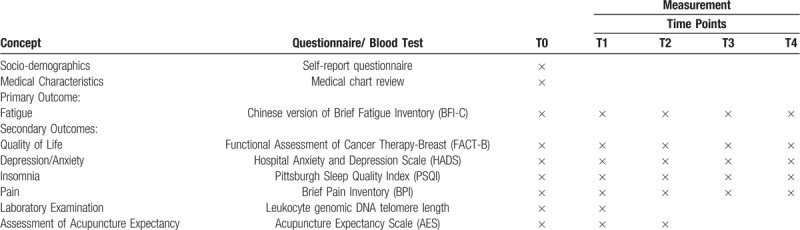
Data collection time points of all outcome measures.

#### 3.7.1 Primary Outcome

Our primary outcome is the fatigue score measured by the Chinese version of the Brief Fatigue Inventory (BFI-C).^[30,31]^ The scale score has excellent internal consistency of 0.96. The indicators of the scale assessment include the current level of fatigue; the fatigue level within the past 24 hours; and the impact of CRF on physical activities, emotions, ability to walk, ability to work, relationships with others, and enjoyment of life within the past 24 hours. The BFI-C uses 10-point numeric descriptions: scores of 1 to 3 represent mild levels of fatigue, scores of 4 to 6 represent moderate levels of fatigue, and scores of 7 to 10 represent severe levels of fatigue. The endpoint is the change in fatigue score from Baseline to Week 6 (end of treatment) among the 3 groups.

#### 3.7.2 Secondary outcomes

##### 3.7.2.1 Quality of Life (QOL)

We will use the Chinese version of the Functional Assessment of Cancer Therapy-Breast (FACT-B) to measure quality of life in BCS. The FACT-B is a breast-specific module comprised of the original four subscales of the Functional Assessment for Cancer Therapy - General (FACT-G) scale: physical well-being, functional well-being, emotional well-being, and social/family well-being, adding a breast cancer-specific subscale.^[32]^ It is a 37-item instrument and each question is rated on a 5-point Likert scale. Higher scores represent improved global QOL.^[33]^ The simplified Chinese version of the FACT-B is validated and reliable for Chinese breast cancer patients with a Cronbach's α of >0.80 in most domains except in additional concerns (0.59).^[34]^

##### 3.7.2.2 Depression/Anxiety

We will use the Chinese version of the Hospital Anxiety and Depression Scale (HADS) to measure anxiety and depression symptoms in BCS. The HADS is a brief 14-item instrument, with seven items in each of the anxiety and depression scales scored from 0 to 3, resulting in scale scores ranging from 0 to 21.^[35]^ Established cutoffs are: 0 to 7, not significant; 8 to 10, subclinical; and 11 to 21, clinically significant depression/anxiety. The Chinese version of HADS is a reliable and valid measure of both anxiety and depression in cancer patients with Cronbach α ranging from 0.86 to 0.93.^[36]^

### 3.8 Stress

We will use the Chinese version of 10-item Perceived Stress Scale (PSS-10) to measure stress in BCS. The PSS-10 is a shortened version of the original 14-item version and psychometrically superior to it with a Cronbach's α >0.70 are usually recommended.^[37]^ Each question in this 10-item instrument is rated on a 5-point Likert scale (0 = never to 4 = very often). A higher score indicates greater stress.

### 3.9 Insomnia

We will use the Chinese version of the Pittsburgh Sleep Quality Index (PSQI) to assess sleep disturbance in BCS. The PSQI is a 19-item self-report instrument that includes seven component scores: subjective sleep quality, sleep latency, sleep duration, habitual sleep efficiency, sleep disturbances, sleep medication, and daytime dysfunction.^[38]^

The scores for these components range from 0 (no difficulty) to 3 (severe difficulty), with a higher score denoting poorer sleep quality (range: 0–21). It suggests a global score cut-off of 8 for the presence of sleep disturbance in cancer patients and has a Cronbach α of 0.79.^[39]^

### 3.10 Pain

We will use the Chinese version of the Brief Pain Inventory (BPI) to assess cancer pain. The BPI is an 11-item self-administered questionnaire that includes two main scores: a pain severity score (scored from 0 to 10, ranging from 0 to 40) and a pain interference score (scored from 0 to 10, ranging from 0 to 70).^[40]^ The Chinese version of the BPI is a reliable and valid measure of both the severity and impact of pain in cancer patients, with Cronbach α of 0.894 and 0.915, respectively.^[41]^

### 3.11 Laboratory examination

We will collect 6-mL ethylenediamine tetra-acetic acid tubes of blood from participants at T0, T2. We will process these samples and store them into −80°C feezer within 24 hours of collection. Then we will prepare the blood samples to evaluate leukocyte genomic DNA telomere length and cortisol.

### 3.12 Assessment of treatment expectancy

We will modify the Chinese version of the Acupuncture Expectancy Scale (AES) to assess response expectancy of laser moxibustion at T0, T1, and T2 (end of intervention). The AES is a 4-item instrument designed to assess outcome expectancy in acupuncture clinical trials, each with a 5-point Likert scale ranging from 1 to 5. The scores range between 4 and 20, with a higher score indicating greater expectancy. It has demonstrated reliability (Cronbach α of 0.82) and validity and is positively correlated with patient self-reported efficacy and satisfaction.^[42]^

### 3.13 Assessment of adverse events

We will monitor patients for adverse events (AEs) during the study period and record any incidents in the case report forms. At the post-intervention (T2), a research assistant will collect information from patients about whether they have experienced or are experiencing AEs during the intervention (ILM or SILM) period. The principal investigator or treating clinician will assign the attribution of the AEs.

### 3.14 Criteria for discontinuation

Every effort will be made to retain patients in the trial and to minimize withdrawals. However, the trial will be discontinued on condition that any serious adverse events happen. Additionally, patients may request to be withdrawn from this study at any time without any reason.

### 3.15 Statistical analysis and sample size calculation

Standard descriptive statistics will be used to analyze baseline participant characteristics. We will produce summary statistics such as means, medians, standard deviations, and ranges for measured variables. We will tabulate frequencies for categorical and ordinal variables. We will use graphical methods extensively to examine distributions, identify potentially influential points, and guide data transformations if warranted. For continuous variables with markedly non-normal or skewed distributions, appropriate transformation may be required, such as natural logarithms, which we will apply as necessary and appropriate.^[43]^ In the sections below, we will assume, when discussing particular outcomes, that the appropriately transformed variables will be used. We will perform analysis according to the intention-to-treat (ITT) principle (i.e., subjects will be analyzed according to the treatment group to which they will be randomly allocated regardless of drop-out).

To determine the effects of ILM on fatigue, we will use mixed effects models.^[44]^ This statistical procedure takes into account within-subject correlations from repeated measurements in the same subjects and allows estimation of between-group difference without necessitating that the last observation is carried forward or exclusion of participants with missing data. Tests of ITT differences between intervention arms with respect to change in BFI-C will be based on time-intervention interactions in the mixed-effects models. Although randomization theoretically balances the potential confounders between treatment groups, occasional unequal distribution can be seen. Should this happen, the effect of confounding will be evaluated by including the potential confounders as co-variates in the models.^[43]^ For secondary outcomes, we will use similar analytical strategies for co-morbid symptoms. To evaluate the long-term durability of treatment effect, we will display long-term data descriptively. It is possible that the long-term outcome may display different patterns from short-term therapeutic effect. If this occurs, we may need to create piece-wise linear mixed-effects models to fit the data and test for statistical significance on outcome change among treatment groups.

We have based our sample size calculation on our preliminary study. We plan to enroll and randomize 140 participants (2:2:1) to ILM, SILM, and WLC. Assuming a 20% attrition rate, we will have 45 participants in each of the ILM and SILM groups and 22 participants in the WLC group who will provide evaluable outcomes. In our previous study, we found that ILM produced a greater reduction in BFI-C intensity at the end of intervention (Week 4 from baseline) at a magnitude of 0.60 SD (Standard Deviation) as compared to SILM. Given an alpha of 5%, with 45 subjects in each of the ILM and SILM groups, we will be able to detect a between-group difference of reduction in BFI-C of 0.60 SD with 80% power. With 45 subjects each in the ILM and SILM groups and 22 participants in the WLC group, a power of 80% and two-sided alpha of 0.05, we will be able to detect an effect size of 0.71 SD (i.e., difference of reduction in BFI-C) between ILM and WLC or SILM and WLC groups. This is a conservative way to estimate sample size since the longitudinal analysis using all repeated measures with the mixed-effects model specified in the analysis plan will provide higher power than a *t* test.

### 3.16 Ethics and dissemination

This trial will be conducted in accordance with the latest revision of the *Declaration of Helsinki* governing standards for good clinical practice. Patient confidentiality will be guaranteed because the data will be de-identified. The results of the clinical trial will be published independently and transparently, regardless of the results.

## 4 Discussion

Cancer-related fatigue is the most common and debilitating symptom experienced by many cancer survivors. CRF often co-occurs with other symptoms such as pain, sleep disturbances, anxiety, and depression that follow a similar time course in relation to cancer or its treatment. NCCN clinical guidelines put a major emphasis on the various aspects of the prevention and treatment of CRF, including management of comorbidities (e.g., pain, insomnia, anxiety, and depression).^[29]^ In particular, some research has shown a significant link between fatigue, joint pain, and insomnia in breast cancer patients.^[45,46]^ In addition, there is growing evidence of acupuncture's impact on cancer survivors with fatigue, anxiety, depression, insomnia, and pain.^[17,18]^ In our preliminary study, infrared laser moxibustion appeared to be safe and efficacious for improving CRF.^[25]^ Hence, we conduct this trial to confirm that infrared laser moxibustion will both reduce fatigue and result in greater improvement in co-morbidities.

Telomeres cap the ends of linear chromosomes and play a role in maintaining genomic stability. Telomere length (TL) is increasingly being examined as a biomarker of accumulated cellular damage and human aging, and is associated with quality of life in cancer patients.^[47]^ Telomere shortening occurs via normal aging, but can be accelerated through exposure to oxidative stress, which plays a major role in the pathophysiology of several chronic inflammatory diseases.^[48,49]^ Immune cell telomere shortness is linked to many chronic disease states and earlier mortality. TL, which may be regarded as a susceptible biomarker of psychosocial influences (particularly stress), and is associated with fatigue.^[50,51]^ TL shortening can be caused by stress, while a decompression intervention can affect TL in cancer patients.^[52–54]^ Therefore, it is hypothesized that telomere length reduces at a faster rate during periods of stress and, therefore, assessment of TL might be a useful biomarker in the progression of CRF.

The strengths of this study come from our combination of rigorous study design and a novel noninvasive and painless moxibustion therapy. To our knowledge, this is the first trial to evaluate the definitive efficacy of infrared laser moxibustion and its durable treatment of CRF in survivors of breast cancer. Outcome expectancy has long been considered an important predictor of treatment outcomes and has gained important consideration in acupuncture research in recent years.^[55]^ The AES has also been validated in breast cancer survivors and is sensitive to change over time in response to acupuncture treatment.^[56]^ This trial is the first to assess response expectancy of laser moxibustion to fatigue among breast cancer survivors. We will be able to explore how pre-treatment expectancy influence therapeutic response in real and sham laser moxibustion interventions. Findings from subjective data (various questionnaires) and objective data (e.g., telomere length) will enhance our understanding of the overall treatment effect and mechanisms of ILM.

Despite the novelty and strengths of this trial, it is not without limitations. First, it is a partial double-blind trial since we include a waitlist control group. Our previous randomized, double-blind, placebo-controlled pilot trial preliminarily demonstrated the efficacy of infrared laser moxibustion in CRF. In this trial, we have added a usual care group to estimate the overall effect of laser moxibustion for CRF. In addition, this study is conducted in China so its generalizability beyond the Chinese population is unknown. If the findings of this trial are positive, we then plan to conduct a follow-up study in populations outside of China.

Despite these limitations, this trial will enhance our understanding of the specific efficacy and overall treatment effect of ILM for cancer-related fatigue and co-morbid symptoms. The results of our study have the potential to improve symptom control and quality of life for millions of breast cancer survivors worldwide.

## Author contributions

**Data curation:** Junchao Chen, Qing Li.

**Formal analysis:** Xuefen Chen.

**Funding acquisition:** Huijuan Mao, Jun J. Mao.

**Investigation:** Xubo Shen, Ling Zhao.

**Methodology:** Huijuan Mao, Jun J. Mao, Xueyong Shen.

**Project administration:** Xueyong Shen.

**Software:** Junchao Chen.

**Supervision:** Jianzi Wei.

**Writing – original draft:** Huijuan Mao, Xueyong Shen.

**Writing – review & editing:** Huijuan Mao, Jun J. Mao, Junchao Chen, Qing Li, Xuefen Chen, Xubo Shen, Ling Zhao, Jianzi Wei, Xueyong Shen.
